# Supplementary material for: Associations of U.S. state-level COVID-19 policies intensity with cannabis sharing behaviors in 2020
Source: Harm Reduct J. 2024 Apr 16;21:82. doi: 10.1186/s12954-024-00987-y (PMC11020667; doi:10.1186/s12954-024-00987-y)
Supplement: Supplementary file 1 — Supplementary Material 1 [file 12954_2024_987_MOESM1_ESM.docx]

**Table Supplement 1** COVID-19 policy score coding by policy type

|  | **Score** | | |  |  |  |
| --- | --- | --- | --- | --- | --- | --- |
| **Policy** | **5** | **4** | **3** | **2** | **1** | **0** |
| **Stay at home order** | •Statewide •New stay at home order | •High-risk groups •Rolled back to high-risk groups |  |  |  | •Lifted •No state policy |
| **Non-essential business closures** |  | •Some non-essential businesses permitted to reopen | •Some non-essential business permitted to reopen with reduced capacity •New business closures or limits | •All non-essential businesses permitted to reopen with reduced capacity |  | •All non-essential businesses permitted to reopen •No state policy |
| **Large gathering ban** |  | •All gatherings prohibited | •>10 people prohibited •Expanded to new limit below 25 •Expanded to new limit of 25 •Expanded to new limit of 25 or fewer •New limit on large gatherings in place | •Expanded to new limit above 25 |  | •Lifted •Other •No state policy |
| **Restaurant limits** |  | •Closed except for takeout/delivery •Newly closed to dine-in service | •Reopened to dine-in service with capacity limits •Limited dine-in service •New capacity limits •New service limits |  |  | •Reopened to dine-in service •No state policy |
| **Bar closures** | •Closed •Newly closed | •New service limits |  |  |  | •Reopened •No state policy |
| **Face covering requirement** | •Required for general public | •Required for certain employees; Allows local officials to require for general public •Required for certain employees •Allows local officials to require for general public |  |  |  | •No state policy |

**Table Supplement 2.** COVID-19 Policy, State Demographic Characteristics, Cannabis State Regulation, COVID-19 Infection Prevalence per 100,000 persons by State

| **State** | **Average COVID-19 Policy Score (June to August)^a^** | **COVID-19 Policy Score June ^a^** | **COVID-19 Policy Score July ^a^** | | **COVID-19 Policy Score August ^a^** | **State Percent Urban** | **State Median  Age** | **Cannabis Regulation** | **COVID-19 Infection Prevalence as of May 24^b^** |
| --- | --- | --- | --- | --- | --- | --- | --- | --- | --- |
| Alabama | 5.3 | 3 | | 6 | 7 | 59.0 | 39.4 | Unregulated | 292.1 |
| Alaska | 2.7 | 0 | | 4 | 4 | 66.0 | 35.0 | Fully Regulated | 55.8 |
| Arizona | 14.5 | 7.5 | | 18 | 18 | 89.8 | 38.2 | Medical Only | 229.0 |
| Arkansas | 8.0 | 9 | | 7 | 8 | 56.2 | 38.5 | Medical Only | 196.6 |
| California | 25.3 | 24 | | 26 | 26 | 95.0 | 37.0 | Fully Regulated | 241.0 |
| Colorado | 20.2 | 19.5 | | 22 | 19 | 86.2 | 37.1 | Fully Regulated | 418.7 |
| Connecticut | 16.5 | 13.5 | | 18 | 18 | 88.0 | 41.1 | Medical Only | 1122.3 |
| Delaware | 16.2 | 13.5 | | 18 | 17 | 83.3 | 41.1 | Medical Only | 889.8 |
| District of Columbia | 16.5 | 13.5 | | 18 | 18 | 100.0 | 34.2 | Fully Regulated | 1176.1 |
| Florida | 12.3 | 9 | | 14 | 14 | 91.2 | 42.5 | Medical Only | 236.2 |
| Georgia | 13.2 | 19.5 | | 10 | 10 | 75.1 | 37.1 | Unregulated | 400.5 |
| Hawaii | 16.5 | 22.5 | | 16 | 11 | 91.9 | 39.6 | Medical Only | 44.2 |
| Idaho | 6.8 | 10.5 | | 5 | 5 | 70.6 | 36.9 | Unregulated | 142.8 |
| Illinois | 13.7 | 15 | | 13 | 13 | 88.5 | 38.6 | Fully Regulated | 860.9 |
| Indiana | 11.7 | 12 | | 11 | 12 | 72.4 | 37.9 | Unregulated | 462.4 |
| Iowa | 8.0 | 12 | | 6 | 6 | 64.0 | 38.5 | Unregulated | 540.7 |
| Kansas | 6.3 | 9 | | 5 | 5 | 74.2 | 37.1 | Unregulated | 306.5 |
| Kentucky | 16.3 | 15 | | 14 | 20 | 58.4 | 39.1 | Unregulated | 190.2 |
| Louisiana | 15.2 | 13.5 | | 13 | 19 | 73.2 | 37.5 | Medical Only | 798.0 |
| Maine | 21.3 | 21 | | 24 | 19 | 38.7 | 45.0 | Fully Regulated | 150.8 |
| Maryland | 12.8 | 13.5 | | 14 | 11 | 87.2 | 39.1 | Medical Only | 749.7 |
| Massachusetts | 21.5 | 22.5 | | 21 | 21 | 92.0 | 39.6 | Fully Regulated | 1318.3 |
| Michigan | 18.0 | 15 | | 19 | 20 | 74.6 | 39.9 | Fully Regulated | 542.6 |
| Minnesota | 14.7 | 15 | | 14 | 15 | 73.3 | 38.3 | Medical Only | 360.5 |
| Mississippi | 13.7 | 12 | | 12 | 17 | 49.4 | 38.0 | Unregulated | 447.8 |
| Missouri | 1.0 | 3 | | 0 | 0 | 70.4 | 38.9 | Medical Only | 197.5 |
| Montana | 9.8 | 10.5 | | 7 | 12 | 55.9 | 40.1 | Medical Only | 44.2 |
| Nebraska | 9.7 | 9 | | 10 | 10 | 73.1 | 36.8 | Unregulated | 618.6 |
| Nevada | 17.3 | 21 | | 18 | 13 | 94.2 | 38.3 | Fully Regulated | 253.9 |
| New Hampshire | 13.7 | 21 | | 10 | 10 | 60.3 | 43.1 | Medical Only | 301.2 |
| New Jersey | 19.8 | 22.5 | | 18 | 19 | 94.7 | 40.1 | Medical Only | 1662.2 |
| New Mexico | 21.2 | 22.5 | | 23 | 18 | 77.4 | 38.4 | Medical Only | 327.9 |
| New York | 19.2 | 22.5 | | 18 | 17 | 87.9 | 39.2 | Medical Only | 1789.6 |
| North Carolina | 17.2 | 13.5 | | 19 | 19 | 66.1 | 39.1 | Unregulated | 223.8 |
| North Dakota | 0.0 | 0 | | 0 | 0 | 59.9 | 35.3 | Medical Only | 310.4 |
| Ohio | 12.8 | 13.5 | | 10 | 15 | 77.9 | 39.5 | Medical Only | 270.4 |
| Oklahoma | 10.5 | 19.5 | | 6 | 6 | 66.2 | 36.9 | Medical Only | 152.5 |
| Oregon | 17.7 | 18 | | 16 | 19 | 81.0 | 39.6 | Fully Regulated | 92.7 |
| Pennsylvania | 15.5 | 16.5 | | 12 | 18 | 78.7 | 40.8 | Medical Only | 548.1 |
| Rhode Island | 13.8 | 13.5 | | 14 | 14 | 90.7 | 40.1 | Medical Only | 1281.7 |
| South Carolina | 9.5 | 10.5 | | 4 | 14 | 66.3 | 39.9 | Unregulated | 197.3 |
| South Dakota | 0.0 | 0 | | 0 | 0 | 56.7 | 37.4 | Unregulated | 514.6 |
| Tennessee | 5.0 | 3 | | 6 | 6 | 66.4 | 39.0 | Unregulated | 291.0 |
| Texas | 16.7 | 12 | | 19 | 19 | 84.7 | 35.0 | Unregulated | 192.0 |
| Utah | 5.0 | 3 | | 6 | 6 | 90.6 | 31.3 | Medical Only | 256.5 |
| Vermont | 16.0 | 21 | | 13 | 14 | 38.9 | 43.0 | Fully Regulated | 148.7 |
| Virginia | 16.3 | 21 | | 14 | 14 | 75.5 | 38.6 | Unregulated | 419.9 |
| Washington | 15.3 | 12 | | 17 | 17 | 84.1 | 37.8 | Fully Regulated | 257.3 |
| West Virginia | 13.5 | 13.5 | | 13 | 14 | 48.7 | 42.9 | Medical Only | 98.1 |
| Wisconsin | 1.7 | 0 | | 0 | 5 | 70.2 | 39.8 | Unregulated | 259.2 |
| Wyoming | 5.5 | 4.5 | | 6 | 6 | 64.8 | 38.4 | Unregulated | 145.3 |

^a^ COVID-19 Policy is a calculated form a score that ranges from 0–30

^b^ COVID-19 infection are state population level prevalence's per 100,000 persons

**Table Supplement 3**. Spearman's correlation coefficient for pairwise correlations between state-level COVID-19 policies (June 2020)

|  | **Stay at home**  **order** | **Non-essential business closure** | **Large gathering ban** | **Restaurant limits** |
| --- | --- | --- | --- | --- |
| Stay at home order | 1.00 |  |  |  |
| Non-essential business closure | **0.39*** | 1.00 |  |  |
| Large gathering ban | **0.36*** | 0.25 | 1.00 |  |
| Restaurant limits | 0.26 | **0.62*** | **0.52*** | 1.00 |

Computed for 50 states including the District of Colombia for policies in June 2020

*p < 0.05

**Table Supplement 4**. Spearman's correlation coefficient for pairwise correlations between state-level COVID-19 policies (July 2020)

|  | **Stay at home**  **order** | **Non-essential business closure** | **Large gathering ban** | **Restaurant limits** | **Bar closures** | **Face cover requirement** |
| --- | --- | --- | --- | --- | --- | --- |
| Stay at home order | 1.00 |  |  |  |  |  |
| Non-essential business closure | **0.32*** | 1.00 |  |  |  |  |
| Large gathering ban | **0.29*** | **0.45*** | 1.00 |  |  |  |
| Restaurant limits | -0.04 | **0.56*** | **0.37*** | 1.00 |  |  |
| Bar closures | 0.21 | **0.44*** | **0.30*** | **0.36*** | 1.00 |  |
| Face cover requirement | 0.22 | **0.44*** | **0.52*** | 0.24 | **0.38*** | 1.00 |

Computed for 50 states including the District of Colombia for policies in July 2020

*p < 0.05

**Table Supplement 5.** Spearman’s correlation coefficient for pairwise correlations between state-level COVID-19 policies (August 2020)

|  | Stay at home  order | Non-essential business closure | Large gathering ban | Restaurant limits | Bar closures | Face cover requirement |
| --- | --- | --- | --- | --- | --- | --- |
| Stay at home order | 1.00 |  |  |  |  |  |
| Non-essential business closure | 0.06 | 1.00 |  |  |  |  |
| Large gathering ban | 0.22 | **0.42*** | 1.00 |  |  |  |
| Restaurant limits | -0.04 | **0.61*** | **0.33*** | 1.00 |  |  |
| Bar closures | 0.06 | **0.53*** | **0.46*** | **0.37*** | 1.00 |  |
| Face cover requirement | 0.10 | **0.40*** | **0.52*** | 0.27 | **0.34*** | 1.00 |

Computed for 50 states including the District of Colombia for policies in August 2020

*p < 0.05
